# Supplementary material for: The Systems Biology Research Tool: evolvable open-source software
Source: BMC Syst Biol. 2008 Jun 29;2:55. doi: 10.1186/1752-0509-2-55 (PMC2446383; doi:10.1186/1752-0509-2-55)
Supplement: Additional file 1 — SBRT Archive. An archive of the current version of the Systems Biology Research Tool. [file 1752-0509-2-55-S1.zip › sbrt-1.4.0/doc/developers_guide/api/sbrt/kernel/math/class-use/Interval.html]

Uses of Class sbrt.kernel.math.Interval


|  |  |  |
| --- | --- | --- |
| **Overview** \| **Package** \| **Class** \| **Use** \| **Tree** \| **Deprecated** \| **Index** \| **Help** | |  |
| PREV   NEXT | **FRAMES**    **NO FRAMES**     **All Classes** |  |


---


## **Uses of Class sbrt.kernel.math.Interval**

| Packages that use Interval | |
| --- | --- |
| **sbrt.ext.cplex** | Provides a class that allows communication with CPLEX. |
| **sbrt.ext.glpk** | Provides a class that allows communication with GLPK. |
| **sbrt.kernel.fba** | Provides classes and interfaces for creating objects relevant to Flux Balance Analysis. |
| **sbrt.kernel.fba.analysis** | Provides classes and interfaces for analyzing Flux Balance Analysis data. |
| **sbrt.kernel.fba.comp** | Provides classes and interfaces for performing Flux Balance Analysis computations. |
| **sbrt.kernel.fba.proc** | Provides classes and interfaces for executing Flux Balance Analysis processes. |
| **sbrt.kernel.math** | Provides classes and interfaces for creating objects relevant to mathematics. |
| **sbrt.kernel.math.algebra** | Provides classes and interfaces for creating objects relevant to algebra. |
| **sbrt.kernel.math.geom** | Provides classes and interfaces for creating objects relevant to computational geometry. |
| **sbrt.kernel.math.optimization** | Provides classes and interfaces for creating objects relevant to solving optimization problems. |
| **sbrt.kernel.utilities.proc** | Provides classes and interfaces for performing utility processes. |
| **sbrt.shell.io.fba** | Provides classes and interfaces for reading and writing files relevant to Flux Balance Analysis. |
| **sbrt.shell.mng.fba** | Provides classes and interfaces for creating Flux Balance Analysis process managers. |
| **sbrt.shell.text.fba** | Provides classes and interfaces for defining formats for objects relevant to Flux Balance Analysis. |
| **sbrt.shell.text.math** | Provides classes and interfaces for defining formats for mathematical objects. |

Uses of Interval in sbrt.ext.cplex

| Methods in sbrt.ext.cplex with parameters of type Interval | |
| --- | --- |
| `<T extends C> void` | `CplexSolver.setColumnConstraint(T column, Interval c)`             Sets the constraint on the provided column variable. |
| `void` | `CplexSolver.setConstraint(LinearComb<? extends C> linComb, Interval c)`             Sets a constraint on the provided mathematical expression of column variables. |
| `void` | `CplexSolver.setConstraint(MathExpr<? extends C> expression, Interval c)`             Sets a constraint on the provided mathematical expression of column variables. |
| `void` | `CplexSolver.setConstraint(QuadraticExpr<? extends C> expr, Interval c)`             Sets a constraint on the provided mathematical expression of column variables. |
| `<T extends R> void` | `CplexSolver.setRowConstraint(T row, Interval c)`             Sets the constraint for the provided row variable. |

| Method parameters in sbrt.ext.cplex with type arguments of type Interval | |
| --- | --- |
| `void` | `CplexSolver.setColumnConstraints(java.util.Map<? extends C,Interval> constraints)`             Sets the constraints for the provided column variables. |
| `void` | `CplexSolver.setConstraints(java.util.Map<? extends MathExpr<? extends C>,Interval> exprConstraints)`             Sets constraints on the provided mathematical expressions of column variables. |
| `void` | `CplexSolver.setRowConstraints(java.util.Map<? extends R,Interval> rowConstraints)`             Sets the constraints for the provided row variables. |

Uses of Interval in sbrt.ext.glpk

| Methods in sbrt.ext.glpk with parameters of type Interval | |
| --- | --- |
| `<Y extends C> void` | `GlpkSolver.setColumnConstraint(Y column, Interval c)`             Sets the constraint on the provided column variable. |
| `void` | `GlpkSolver.setConstraint(LinearComb<? extends C> expression, Interval c)`             Sets a constraint on the provided mathematical expression of column variables. |
| `void` | `GlpkSolver.setConstraint(MathExpr<? extends C> expression, Interval c)`             Sets a constraint on the provided mathematical expression of column variables. |
| `<X extends R> void` | `GlpkSolver.setRowConstraint(X row, Interval c)`             Sets the constraint for the provided row variable. |

| Method parameters in sbrt.ext.glpk with type arguments of type Interval | |
| --- | --- |
| `void` | `GlpkSolver.setColumnConstraints(java.util.Map<? extends C,Interval> columnConstraints)`             Sets the constraints for the provided column variables. |
| `void` | `GlpkSolver.setConstraints(java.util.Map<? extends MathExpr<? extends C>,Interval> expressionConstraints)`             Sets constraints on the provided mathematical expressions of column variables. |
| `void` | `GlpkSolver.setRowConstraints(java.util.Map<? extends R,Interval> rowConstraints)`             Sets the constraints for the provided row variables. |

Uses of Interval in sbrt.kernel.fba

| Fields in sbrt.kernel.fba declared as Interval | |
| --- | --- |
| `static Interval` | `IrrevRxnFluxomeV1.OFF`             The constraint used by default for all source reactions: [0, 0]. |
| `static Interval` | `IrrevRxnFluxomeV1.ON`             The constraint used by default for all internal and sink reactions: [0, Infinity). |

| Methods in sbrt.kernel.fba that return Interval | |
| --- | --- |
| `Interval` | `FluxConstraints.getConstraint(MathExpr<java.lang.String> rxnExpr)`             Returns the constraint of the provided mathematical expression of reaction names. |
| `Interval` | `FluxConstraints.getConstraint(java.lang.String rxnName)`             Returns the flux constraint for the provided reaction. |
| `Interval` | `Fluxome.getFluxConstraint(java.lang.String reactionName)`             Returns the flux constraint corresponding to the provided reaction. |
| `Interval` | `IrrevRxnFluxomeV1.getFluxConstraint(java.lang.String reactionName)`             Returns the flux constraint for the provided reaction. |
| `Interval` | `Fluxome.getRxnExprConstraint(MathExpr<java.lang.String> rxnNameExpr)`             Returns the constraint corresponding to the provided mathematical expression of reaction names. |
| `Interval` | `IrrevRxnFluxomeV1.getRxnExprConstraint(MathExpr<java.lang.String> rxnNameExpr)`             Returns the constraint associated with the expression provided. |

| Methods in sbrt.kernel.fba that return types with arguments of type Interval | |
| --- | --- |
| `UnmodifiableMap<MathExpr<java.lang.String>,Interval>` | `FluxConstraints.getExprConstraints()`             Returns the constraints for mathematical expressions of fluxes. |
| `UnmodifiableMap<java.lang.String,Interval>` | `FluxConstraints.getFluxConstraints()`             Returns the flux constraints for single reactions. |
| `java.util.Map<java.lang.String,Interval>` | `IrrevRxnFluxomeV1.getFluxConstraints()`             Returns the constraints on each flux in this fluxome. |

| Methods in sbrt.kernel.fba with parameters of type Interval | |
| --- | --- |
| `boolean` | `Fluxome.isValid(Interval c, java.lang.String rxnName, double tolerance)`             Indicates if the provided interval is a valid flux constraint for the reaction with the provided name. |
| `boolean` | `IrrevRxnFluxomeV1.isValid(Interval c, java.lang.String rxnName, double tolerance)`             Indicates if the provided interval is a valid flux constraint for the reaction having the provided name. |

Constructor parameters in sbrt.kernel.fba with type arguments of type Interval

`FluxConstraints(java.util.Map<java.lang.String,Interval> fluxConstraints)`
  
          Constructs a set of FBA constraints from the provided argument.

`FluxConstraints(java.util.Map<java.lang.String,Interval> fluxConstraints,
java.util.Map<? extends MathExpr<java.lang.String>,Interval> exprConstraints)`
  
          Constructs a set of FBA constraints from the provided arguments.

`FluxConstraints(java.util.Map<java.lang.String,Interval> fluxConstraints,
java.util.Map<? extends MathExpr<java.lang.String>,Interval> exprConstraints)`
  
          Constructs a set of FBA constraints from the provided arguments.

Uses of Interval in sbrt.kernel.fba.analysis

| Method parameters in sbrt.kernel.fba.analysis with type arguments of type Interval | |
| --- | --- |
| `java.util.Set<java.lang.String>` | `FluxIntervalAnalyzer.getAlwaysActive(java.util.Map<java.lang.String,Interval> fluxIntervals, double zeroCutoff)`             Returns the always-active fluxes in the provided map. |
| `java.util.Set<java.lang.String>` | `FluxIntervalAnalyzer.getNeverActive(java.util.Map<java.lang.String,Interval> fluxIntervals, double zeroCutoff)`             Returns the never-active fluxes in the provided map. |
| `java.util.Set<java.lang.String>` | `FluxIntervalAnalyzer.getPotentiallyActive(java.util.Map<java.lang.String,Interval> fluxIntervals, double zeroCutoff)`             Returns the potentially-active fluxes in the provided map. |
| `int` | `FluxIntervalAnalyzer.lowerBoundsEqualZero(java.util.Collection<Interval> intervals, double zeroCutoff)`             Returns the number of provided intervals whose lower bound equals zero. |
| `int` | `FluxIntervalAnalyzer.lowerBoundsEqualZero(java.util.Map<?,Interval> intervals, double zeroCutoff)`             Returns the number of provided intervals whose lower bound equals zero. |
| `int` | `FluxIntervalAnalyzer.lowerBoundsGreaterThanZero(java.util.Collection<Interval> intervals, double zeroCutoff)`             Returns the number of provided intervals whose lower bounds are greater than zero. |
| `int` | `FluxIntervalAnalyzer.lowerBoundsGreaterThanZero(java.util.Map<?,Interval> intervals, double zeroCutoff)`             Returns the number of provided intervals whose lower bounds are greater than zero. |
| `int` | `FluxIntervalAnalyzer.rangesEqualZero(java.util.Collection<Interval> intervals, double zeroCutoff)`             Returns the number of provided intervals whose ranges equal zero. |
| `int` | `FluxIntervalAnalyzer.rangesEqualZero(java.util.Map<?,Interval> intervals, double zeroCutoff)`             Returns the number of provided intervals whose ranges equal zero. |
| `int` | `FluxIntervalAnalyzer.rangesGreaterThanZero(java.util.Collection<Interval> intervals, double zeroCutoff)`             Returns the number of provided intervals whose ranges are greater than zero. |
| `int` | `FluxIntervalAnalyzer.rangesGreaterThanZero(java.util.Map<?,Interval> intervals, double zeroCutoff)`             Returns the number of provided intervals whose ranges are greater than zero. |
| `int` | `FluxIntervalAnalyzer.upperBoundsEqualZero(java.util.Collection<Interval> intervals, double zeroCutoff)`             Returns the number of provided intervals whose upper bounds equal zero. |
| `int` | `FluxIntervalAnalyzer.upperBoundsEqualZero(java.util.Map<?,Interval> intervals, double zeroCutoff)`             Returns the number of provided intervals whose upper bounds equal zero. |
| `int` | `FluxIntervalAnalyzer.upperBoundsGreaterThanZero(java.util.Collection<Interval> intervals, double zeroCutoff)`             Returns the number of provided intervals whose upper bounds are greater than zero. |
| `int` | `FluxIntervalAnalyzer.upperBoundsGreaterThanZero(java.util.Map<?,Interval> intervals, double zeroCutoff)`             Returns the number of provided intervals whose upper bounds are greater than zero. |

Uses of Interval in sbrt.kernel.fba.comp

| Methods in sbrt.kernel.fba.comp that return Interval | |
| --- | --- |
| `Interval` | `FbaOptComputer.getColExprConstraint(MathExpr<?> columnVariableExpr)`             Returns the current constraint on the provided expression. |
| `Interval` | `FbaOptComputer.getColumnConstraint(java.lang.Object columnVariable)`             Returns the current constraint on the provided column variable. |
| `Interval` | `RxnDeletionComputer.getFluxConstraint(java.lang.String rxnName)`             Returns the flux constraint of the reaction with the provided name. |
| `Interval` | `InitialPointComputer.getFluxConstraint(java.lang.String rxnName)`             Returns the current flux constraint on the reaction with the provided name. |
| `Interval` | `FluxCvComputer.getFluxConstraint(java.lang.String rxnName)`             Returns the current flux constraint on the reaction with the provided name. |
| `Interval` | `CdHarFluxVectorComputer.getFluxConstraint(java.lang.String rxnName)`             Returns the current flux constraint on the reaction with the provided name. |
| `Interval` | `AbstractFluxIntervalComputer.getFluxConstraint(java.lang.String rxnName)`             Returns the current flux constraint on the reaction with the provided name. |
| `Interval` | `FbaOptComputer.getFluxConstraint(java.lang.String reactionName)`             Returns the current flux constraint on the reaction with the provided name. |
| `Interval` | `FastMsFluxRangeComputer.getFluxConstraint(java.lang.String rxnName)`             Returns the current flux constraint on the reaction with the provided name. |
| `protected  Interval` | `AbstractFluxIntervalComputer.getInterval(java.lang.String rxn, Interval fluxConstraint, double computedMin, double computedMax)`             Returns the provided minimum and maximum fluxes as an interval. |
| `Interval` | `FbaOptComputer.getRowConstraint(java.lang.Object rowVariable)`             Returns the current constraint on the provided row variable. |
| `Interval` | `RxnDeletionComputer.getRxnExprConstraint(MathExpr<java.lang.String> rxnNameExp)`             Returns the constraint on the provided mathematical expression of fluxes. |
| `Interval` | `InitialPointComputer.getRxnExprConstraint(MathExpr<java.lang.String> rxnNameExp)`             Returns the current constraint on the provided expression. |
| `Interval` | `FluxCvComputer.getRxnExprConstraint(MathExpr<java.lang.String> rxnNameExp)`             Returns the current constraint on the provided expression. |
| `Interval` | `CdHarFluxVectorComputer.getRxnExprConstraint(MathExpr<java.lang.String> rxnNameExp)`             Returns the current constraint on the provided expression. |
| `Interval` | `AbstractFluxIntervalComputer.getRxnExprConstraint(MathExpr<java.lang.String> rxnNameExp)`             Returns the current constraint on the provided expression. |
| `Interval` | `FbaOptComputer.getRxnExprConstraint(MathExpr<java.lang.String> rxnNameExpr)`             Returns the current constraint on the provided expression. |
| `Interval` | `FastMsFluxRangeComputer.getRxnExprConstraint(MathExpr<java.lang.String> rxnNameExp)`             Returns the current constraint on the provided expression. |

| Methods in sbrt.kernel.fba.comp that return types with arguments of type Interval | |
| --- | --- |
| `java.util.Map<MathExpr<?>,Interval>` | `FbaOptComputer.getColExprConstraints()`             Returns all current constraints on mathematical expressions of column variables. |
| `java.util.Map<?,Interval>` | `FbaOptComputer.getColumnConstraints()`             Returns the current constraints for each column variable in the program matrix. |
| `java.util.Map<java.lang.String,Interval>` | `RxnDeletionComputer.getFluxConstraints()`             Returns the set of all constraints on single fluxes. |
| `java.util.Map<java.lang.String,Interval>` | `InitialPointComputer.getFluxConstraints()`             Returns all current flux constraints. |
| `java.util.Map<java.lang.String,Interval>` | `FluxCvComputer.getFluxConstraints()`             Returns all current flux constraints. |
| `java.util.Map<java.lang.String,Interval>` | `CdHarFluxVectorComputer.getFluxConstraints()`             Returns all current flux constraints. |
| `java.util.Map<java.lang.String,Interval>` | `AbstractFluxIntervalComputer.getFluxConstraints()`             Returns all current flux constraints. |
| `java.util.Map<java.lang.String,Interval>` | `FbaOptComputer.getFluxConstraints()`             Returns all current flux constraints for each reaction in the fluxome used by this optimization computer. |
| `java.util.Map<java.lang.String,Interval>` | `FastMsFluxRangeComputer.getFluxConstraints()`             Returns all current flux constraints. |
| `java.util.Map<java.lang.String,Interval>` | `MsFluxIntervalComputer.getIntervals()`             Computes and returns the intervals of every flux in the fluxome used to construct this flux interval computer. |
| `java.util.Map<java.lang.String,Interval>` | `SimpleFluxIntervalComputer.getIntervals()`             Computes and returns the intervals of every flux in the fluxome used to construct this flux interval computer. |
| `java.util.Map<java.lang.String,Interval>` | `FastMsFluxRangeComputer.getIntervals()`             Computes and returns the intervals of every flux in the fluxome used to construct this flux interval computer. |
| `java.util.Map<java.lang.String,Interval>` | `ReverseRxnFluxIntervalComputer.getIntervals()`             Computes and returns the intervals of every flux in the fluxome used to construct this flux interval computer. |
| `java.util.Map<java.lang.String,Interval>` | `FluxIntervalComputer.getIntervals()`             Computes and returns the intervals of every flux in the fluxome used to construct this flux interval computer. |
| `java.util.Map<java.lang.String,Interval>` | `MsFluxIntervalComputer.getIntervals(java.util.Collection<java.lang.String> rxnNames)`             Computes and returns the flux intervals of the reactions with the provided names. |
| `java.util.Map<java.lang.String,Interval>` | `SimpleFluxIntervalComputer.getIntervals(java.util.Collection<java.lang.String> rxnNames)`             Computes and returns the flux intervals of the reactions with the provided names. |
| `java.util.Map<java.lang.String,Interval>` | `FastMsFluxRangeComputer.getIntervals(java.util.Collection<java.lang.String> reactionNames)`             Computes and returns the flux intervals of the reactions with the provided names. |
| `java.util.Map<java.lang.String,Interval>` | `ReverseRxnFluxIntervalComputer.getIntervals(java.util.Collection<java.lang.String> rxnNames)`             Computes and returns the flux intervals of the reactions with the provided names. |
| `java.util.Map<java.lang.String,Interval>` | `FluxIntervalComputer.getIntervals(java.util.Collection<java.lang.String> rxnNames)`             Computes and returns the flux intervals of the reactions with the provided names. |
| `protected  java.util.Map<java.lang.String,Interval>` | `AbstractFluxIntervalComputer.getIntervals(java.util.Map<java.lang.String,java.lang.Double> fluxMinima, java.util.Map<java.lang.String,java.lang.Double> fluxMaxima)`             Returns the provided minima and maxima as intervals. |
| `java.util.Map<?,Interval>` | `FbaOptComputer.getRowConstraints()`             Returns all the current constraints on row variables. |
| `java.util.Map<MathExpr<java.lang.String>,Interval>` | `RxnDeletionComputer.getRxnExprConstraints()`             Returns the set of all constraints on mathematical expressions of fluxes. |
| `java.util.Map<MathExpr<java.lang.String>,Interval>` | `InitialPointComputer.getRxnExprConstraints()`             Returns all current flux expression constraints. |
| `java.util.Map<? extends MathExpr<java.lang.String>,Interval>` | `FluxCvComputer.getRxnExprConstraints()`             Returns all current flux expression constraints. |
| `java.util.Map<LinearComb<java.lang.String>,Interval>` | `CdHarFluxVectorComputer.getRxnExprConstraints()`             Returns all current flux expression constraints. |
| `java.util.Map<MathExpr<java.lang.String>,Interval>` | `AbstractFluxIntervalComputer.getRxnExprConstraints()`             Returns all current flux expression constraints. |
| `java.util.Map<MathExpr<java.lang.String>,Interval>` | `FbaOptComputer.getRxnExprConstraints()`             Returns all current constraints on mathematical expressions of reaction names. |
| `java.util.Map<MathExpr<java.lang.String>,Interval>` | `FastMsFluxRangeComputer.getRxnExprConstraints()`             Returns all current flux expression constraints. |

| Methods in sbrt.kernel.fba.comp with parameters of type Interval | |
| --- | --- |
| `protected  void` | `AbstractCvComputer.checkFluxConstraint(Interval c, java.lang.String rxnName, double tolerance)`             Ensures the flux constraint is valid for the fluxome provided. |
| `protected  void` | `FbaOptComputer.checkFluxConstraint(Interval c, java.lang.String rxnName, double tolerance)`             Ensures the provided constraint is a valid flux constraint in the fluxome used by this optimization computer. |
| `protected  void` | `AbstractCvComputer.checkRxnExprConstraint(Interval c)`             Ensures the expression constraint is valid for the fluxome provided. |
| `protected  void` | `FbaOptComputer.checkRxnExprConstraint(Interval c)`             Ensures the provided constraint is valid for reaction name-expressions for the fluxome used by this optimization computer. |
| `protected  Interval` | `AbstractFluxIntervalComputer.getInterval(java.lang.String rxn, Interval fluxConstraint, double computedMin, double computedMax)`             Returns the provided minimum and maximum fluxes as an interval. |
| `void` | `FbaOptComputer.setColExprConstraint(MathExpr<?> expr, Interval c)`             Changes the constraint on the provided mathematical expression of column variables. |
| `void` | `FbaOptComputer.setColumnConstraint(java.lang.Object columnVar, Interval c)`             Changes the constraint of the provided column variable. |
| `void` | `RxnDeletionComputer.setFluxConstraint(java.lang.String reactionName, Interval c)`             Changes the flux constraint of the reaction with the provided name. |
| `void` | `InitialPointComputer.setFluxConstraint(java.lang.String reactionName, Interval c)`             Changes the flux constraint of the reaction with the provided name. |
| `void` | `FluxCvComputer.setFluxConstraint(java.lang.String reactionName, Interval c)`             Changes the flux constraint of the reaction with the provided name. |
| `void` | `CdHarFluxVectorComputer.setFluxConstraint(java.lang.String reactionName, Interval c)`             Changes the flux constraint of the reaction with the provided name. |
| `void` | `AbstractFluxIntervalComputer.setFluxConstraint(java.lang.String reactionName, Interval c)`             Changes the flux constraint of the reaction with the provided name. |
| `void` | `FbaOptComputer.setFluxConstraint(java.lang.String reactionName, Interval c)`             Changes the flux constraint of the reaction with the provided name. |
| `void` | `FastMsFluxRangeComputer.setFluxConstraint(java.lang.String reactionName, Interval c)`             Changes the flux constraint of the reaction with the provided name. |
| `void` | `FbaOptComputer.setRowConstraint(java.lang.Object rowVariable, Interval c)`             Changes the constraint of the provided row variable. |
| `void` | `CdHarFluxVectorComputer.setRxnExprConstraint(LinearComb<java.lang.String> rxnExpr, Interval c)`             Changes the constraint on the provided linear combination of fluxes. |
| `void` | `RxnDeletionComputer.setRxnExprConstraint(MathExpr<java.lang.String> e, Interval c)`             Changes the constraint on the provided mathematical expression of fluxes. |
| `void` | `InitialPointComputer.setRxnExprConstraint(MathExpr<java.lang.String> e, Interval c)`             Changes the constraint on the provided mathematical expression of fluxes. |
| `void` | `FluxCvComputer.setRxnExprConstraint(MathExpr<java.lang.String> e, Interval c)`             Changes the constraint on the provided mathematical expression of fluxes. |
| `void` | `CdHarFluxVectorComputer.setRxnExprConstraint(MathExpr<java.lang.String> rxnExpr, Interval c)`             Changes the constraint on the provided mathematical expression of fluxes. |
| `void` | `AbstractFluxIntervalComputer.setRxnExprConstraint(MathExpr<java.lang.String> e, Interval c)`             Changes the constraint on the provided mathematical expression of fluxes. |
| `void` | `FbaOptComputer.setRxnExprConstraint(MathExpr<java.lang.String> expr, Interval c)`             Changes the constraint on the provided mathematical expression of fluxes. |
| `void` | `FastMsFluxRangeComputer.setRxnExprConstraint(MathExpr<java.lang.String> e, Interval c)`             Changes the constraint on the provided mathematical expression of fluxes. |

| Method parameters in sbrt.kernel.fba.comp with type arguments of type Interval | |
| --- | --- |
| `void` | `FbaOptComputer.setColExprConstraints(java.util.Map<? extends MathExpr<?>,Interval> exprConstraints)`             Changes the constraints on more than one mathematical expression of column variables. |
| `void` | `FbaOptComputer.setColumnConstraints(java.util.Map<?,Interval> columnConstraints)`             Changes the constraints on more than one column variable. |
| `void` | `InitialPointComputer.setFluxConstraints(java.util.Map<java.lang.String,Interval> fluxConstraints)`             Changes the flux constraints on more than one reaction. |
| `void` | `FluxCvComputer.setFluxConstraints(java.util.Map<java.lang.String,Interval> fluxConstraints)`             Changes the flux constraints on more than one reaction. |
| `void` | `AbstractCvComputer.setFluxConstraints(java.util.Map<java.lang.String,Interval> fluxConstraints)`             Changes the flux constraints on more than one reaction. |
| `void` | `AbstractFluxIntervalComputer.setFluxConstraints(java.util.Map<java.lang.String,Interval> fluxConstraints)`             Changes the flux constraints on more than one reaction. |
| `void` | `FbaOptComputer.setFluxConstraints(java.util.Map<java.lang.String,Interval> fluxConstraints)`             Changes the flux constraints on more than one reaction. |
| `void` | `FastMsFluxRangeComputer.setFluxConstraints(java.util.Map<java.lang.String,Interval> fluxConstraints)`             Changes the flux constraints on more than one reaction. |
| `void` | `FbaOptComputer.setRowConstraints(java.util.Map<?,Interval> rowConstraints)`             Changes the constraints on more than one row variable. |
| `void` | `InitialPointComputer.setRxnExprConstraints(java.util.Map<? extends MathExpr<java.lang.String>,Interval> constraints)`             Changes the constraints on more than one mathematical expression of fluxes. |
| `void` | `FluxCvComputer.setRxnExprConstraints(java.util.Map<? extends MathExpr<java.lang.String>,Interval> constraints)`             Changes the constraints on more than one mathematical expression of fluxes. |
| `void` | `AbstractCvComputer.setRxnExprConstraints(java.util.Map<? extends MathExpr<java.lang.String>,Interval> constraints)`             Changes the constraints on more than one mathematical expression of fluxes. |
| `void` | `AbstractFluxIntervalComputer.setRxnExprConstraints(java.util.Map<? extends MathExpr<java.lang.String>,Interval> constraints)`             Changes the constraints on more than one mathematical expression of fluxes. |
| `void` | `FbaOptComputer.setRxnExprConstraints(java.util.Map<? extends MathExpr<java.lang.String>,Interval> exprConstraints)`             Changes the constraints on more than one mathematical expression of fluxes. |
| `void` | `FastMsFluxRangeComputer.setRxnExprConstraints(java.util.Map<? extends MathExpr<java.lang.String>,Interval> constraints)`             Changes the constraints on more than one mathematical expression of fluxes. |

Uses of Interval in sbrt.kernel.fba.proc

| Methods in sbrt.kernel.fba.proc that return Interval | |
| --- | --- |
| `Interval` | `RandomEnvGeneratorV1.getActive()`             Returns the constraint used to represent an active source flux. |
| `Interval` | `RandomConstraintsGeneratorV1.getActive()`             Returns the constraint used to represent an active flux. |
| `Interval` | `RandomEnvGeneratorV1.getInactive()`             Returns the constraint used to represent an inactive source flux. |
| `Interval` | `RandomConstraintsGeneratorV1.getInactive()`             Returns the constraint used to represent an inactive flux. |

| Methods in sbrt.kernel.fba.proc with parameters of type Interval | |
| --- | --- |
| `void` | `RandomEnvGeneratorV1.setOff(Interval inactive)`             Sets the constraint used to represent an inactive source flux. |
| `void` | `RandomConstraintsGeneratorV1.setOff(Interval inactive)`             Sets the constraint used to represent an inactive flux. |
| `void` | `RandomEnvGeneratorV1.setOn(Interval active)`             Sets the constraint used to represent an active source flux. |
| `void` | `RandomConstraintsGeneratorV1.setOn(Interval active)`             Sets the constraint used to represent an active flux. |

| Method parameters in sbrt.kernel.fba.proc with type arguments of type Interval | |
| --- | --- |
| `void` | `FluxIntervalProcess.run(ResultsManager<java.util.Map<java.lang.String,Interval>> resultsManager)`             Computes the flux ranges of the reactions with the current names and adds them to the provided results manager. |
| `void` | `RandomEnvGeneratorV1.run(ResultsManager<java.util.Map<java.lang.String,Interval>> resultsManager)`             Generates a random environment and adds it to the provided results manager. |
| `void` | `CvFluxIntervalProcess.run(ResultsManager<java.util.Map<java.lang.String,Interval>> resultsManager)`             Runs this process and adds the generated results to the provided results manager via its `ResultsManager.addData(Object)` method. |
| `void` | `RandomConstraintsGeneratorV1.run(ResultsManager<java.util.Map<java.lang.String,Interval>> resultsManager)`             Generates sets of random flux constraints and adds them to the provided results manager. |
| `void` | `FluxPlasticityProcess.setFluxIntervalSource(java.lang.Iterable<java.util.Map<java.lang.String,Interval>> intervalSource)`             Sets the flux interval source. |

Uses of Interval in sbrt.kernel.math

| Methods in sbrt.kernel.math that return Interval | |
| --- | --- |
| `Interval` | `ConstraintViolationException.getBounds()`             Returns the constraints that have been violated. |
| `Interval` | `IntervalComparer.Diff.getInterval1()` |
| `Interval` | `IntervalComparer.Diff.getInterval2()` |

| Methods in sbrt.kernel.math with parameters of type Interval | |
| --- | --- |
| `boolean` | `IntervalEquiv.areEquiv(Interval value1, Interval value2)`             Indicates if the provided intervals are equivalent. |

| Method parameters in sbrt.kernel.math with type arguments of type Interval | |
| --- | --- |
| `<V> java.util.Set<IntervalComparer.Diff<V>>` | `IntervalComparer.getDiffs(java.util.Map<? extends V,Interval> m1, java.util.Map<? extends V,Interval> m2, double tolerance)` |
| `<V> java.util.Set<IntervalComparer.Diff<V>>` | `IntervalComparer.getDiffs(java.util.Map<? extends V,Interval> m1, java.util.Map<? extends V,Interval> m2, double tolerance)` |

Constructors in sbrt.kernel.math with parameters of type Interval

`ConstraintViolationException(java.lang.Object variable,
Interval bounds,
double violatingValue)`
  
          Constructs a new constraint violation exception.

`ConstraintViolationException(java.lang.Object variable,
Interval bounds,
java.lang.Number violatingValue)`
  
          Constructs a new constraint violation exception.

Uses of Interval in sbrt.kernel.math.algebra

Method parameters in sbrt.kernel.math.algebra with type arguments of type Interval

`static

<V> LinearSystemSolution<V>`

`HashLinearSystemSolution.simplify(LinearSystemSolution<V> solution,
java.util.Map<? extends V,Interval> variableBounds,
double tolerance)`
  
          Returns a simplified version of the provided linear system solution
given the variable bounds.

Uses of Interval in sbrt.kernel.math.geom

| Methods in sbrt.kernel.math.geom that return Interval | |
| --- | --- |
| `Interval` | `ParallelHyperplanes.getBounds()`             Returns the bounding values used to define the inequality. |

Method parameters in sbrt.kernel.math.geom with type arguments of type Interval

`static

<V> HashConvexPolytope<V>`

`HashConvexPolytope.make(LinearSystemSolution<V> systemSolution,
java.util.Map<? extends V,Interval> variableBounds,
double tolerance)`
  
          Constructs a new convex poltyope from the provided linear system solution
and variable bounds.

Constructors in sbrt.kernel.math.geom with parameters of type Interval

`ParallelHyperplanes(LinearComb<V> hyperplane,
Interval bounds)`
  
          Constructs a new pair of bounding parallel hyperplanes.

Constructor parameters in sbrt.kernel.math.geom with type arguments of type Interval

`HashConvexPolytopeOld(LinearSystemSolution<V> systemSolution,
java.util.Map<? extends V,Interval> variableBounds,
double tolerance)`
  
          Constructs a new convex poltyope from the provided linear system
solution and variable bounds.

Uses of Interval in sbrt.kernel.math.optimization

| Methods in sbrt.kernel.math.optimization with parameters of type Interval | |
| --- | --- |
| `<Y extends C> void` | `ProgramSolver.setColumnConstraint(Y column, Interval c)`             Sets the constraint on the provided column variable. |
| `void` | `LinearProgramSolver.setConstraint(LinearComb<? extends C> expression, Interval c)`             Sets a constraint on the provided linear combination of column variables. |
| `void` | `ProgramSolver.setConstraint(MathExpr<? extends C> expression, Interval c)`             Sets a constraint on the provided mathematical expression of column variables. |
| `void` | `QuadraticProgramSolver.setConstraint(QuadraticExpr<? extends C> expression, Interval c)`             Sets a constraint on the provided quadratic expression of column variables. |
| `<X extends R> void` | `ProgramSolver.setRowConstraint(X row, Interval c)`             Sets the constraint for the provided row variable. |

| Method parameters in sbrt.kernel.math.optimization with type arguments of type Interval | |
| --- | --- |
| `void` | `ProgramSolver.setColumnConstraints(java.util.Map<? extends C,Interval> columnConstraints)`             Sets the constraints for the provided column variables. |
| `void` | `ProgramSolver.setConstraints(java.util.Map<? extends MathExpr<? extends C>,Interval> expressionConstraints)`             Sets constraints on the provided mathematical expressions of column variables. |
| `void` | `ProgramSolver.setRowConstraints(java.util.Map<? extends R,Interval> rowConstraints)`             Sets the constraints for the provided row variables. |

Uses of Interval in sbrt.kernel.utilities.proc

| Method parameters in sbrt.kernel.utilities.proc with type arguments of type Interval | |
| --- | --- |
| `void` | `IntervalComparison.setIntervalSources(java.lang.Iterable<java.util.Map<V,Interval>> source1, java.lang.Iterable<java.util.Map<V,Interval>> source2)` |
| `void` | `IntervalComparison.setIntervalSources(java.lang.Iterable<java.util.Map<V,Interval>> source1, java.lang.Iterable<java.util.Map<V,Interval>> source2)` |

Uses of Interval in sbrt.shell.io.fba

| Method parameters in sbrt.shell.io.fba with type arguments of type Interval | |
| --- | --- |
| `static FluxConstraints` | `FluxIntervalInputFile.convertConstraints(java.util.Map<LinearComb<java.lang.String>,Interval> intervals)`             Converts the provided intervals into a `FluxConstraints` object. |

Constructor parameters in sbrt.shell.io.fba with type arguments of type Interval

`FluxIntervalInputFile(java.lang.String fileName,
FileFormat fileFormat,
java.util.Set<LinearComb<java.lang.String>> requiredVariables,
MapFormat<LinearComb<java.lang.String>,Interval> mapFormat)`
  
          Constructs a new flux-interval input file.

Uses of Interval in sbrt.shell.mng.fba

| Methods in sbrt.shell.mng.fba that return Interval | |
| --- | --- |
| `static Interval` | `RandomConstraintsManager.getActive(ManagerInput<java.lang.String,java.lang.String> input)`             Parses and returns the value for `RandomConstraintsManager.ACTIVE` in the provided manager input. |
| `static Interval` | `RandomConstraintsManager.getInactive(ManagerInput<java.lang.String,java.lang.String> input)`             Parses and returns the value for `RandomConstraintsManager.INACTIVE` in the provided manager input. |

| Methods in sbrt.shell.mng.fba that return types with arguments of type Interval | |
| --- | --- |
| `static MapInputFile<java.lang.String,Interval>` | `FluxPlasticityManager.getFluxIntervalFile(ManagerInput<java.lang.String,java.lang.String> input, java.io.PrintWriter out, Fluxome fluxome)`             Returns the flux interval input with the name denoted by `FluxPlasticityManager.FLUX_INTERVAL_FILE` in the provided manager input. |
| `static MapInputFile<java.lang.String,Interval>` | `FluxPlasticityManager.getFluxIntervalFile(java.lang.String fileName, Fluxome fluxome)`             Returns the flux interval input file with the provided name. |
| `static ComoFile<java.lang.String,Interval>` | `SimpleFluxIntervalManager.getMultiResultsOutputFile(ManagerInput<java.lang.String,java.lang.String> input, java.io.PrintWriter out, Fluxome fluxome)`             Returns an empty output file that is suitable for storing multiple sets of flux interval results. |
| `static ComoFile<java.lang.String,Interval>` | `SimpleFluxIntervalManager.getMultiResultsOutputFile(java.lang.String fileName, java.util.Set<java.lang.String> headers, Fluxome fluxome)`             Returns an empty output file that is suitable for storing multiple sets of flux interval results. |
| `static MapTextOutputFile<java.lang.String,Interval>` | `RandomConstraintsManager.getOutputFile(ManagerInput<java.lang.String,java.lang.String> input, java.io.PrintWriter out, IrrevRxnFluxome fluxome)`             Returns an empty output file that is suitable for storing multiple sets of flux constraints. |
| `static MapTextOutputFile<java.lang.String,Interval>` | `RandomEnvManager.getOutputFile(ManagerInput<java.lang.String,java.lang.String> input, java.io.PrintWriter out, IrrevRxnFluxome fluxome)`             Returns an empty output file that is suitable for storing multiple environments. |
| `static MapTextOutputFile<java.lang.String,Interval>` | `RandomConstraintsManager.getOutputFile(java.lang.String fileName, FileFormat format, java.util.Set<java.lang.String> headers, IrrevRxnFluxome fluxome)`             Returns an empty output file that is suitable for storing multiple sets of flux constraints. |
| `static MapTextOutputFile<java.lang.String,Interval>` | `RandomConstraintsManager.getOutputFile(java.lang.String fileName, java.util.Set<java.lang.String> headers, IrrevRxnFluxome fluxome)`             Returns an empty output file that is suitable for storing multiple sets of flux constraints. |
| `static RomoFile<java.lang.String,Interval>` | `SimpleFluxIntervalManager.getSingleResultOutputFile(ManagerInput<java.lang.String,java.lang.String> input, java.io.PrintWriter out, Fluxome fluxome)`             Returns an empty output file that is suitable for storing a single set of flux interval results. |
| `static RomoFile<java.lang.String,Interval>` | `SimpleFluxIntervalManager.getSingleResultOutputFile(java.lang.String fileName, java.util.Set<java.lang.String> headers, Fluxome fluxome)`             Returns an empty output file that is suitable for storing a single set of flux interval results. |

| Method parameters in sbrt.shell.mng.fba with type arguments of type Interval | |
| --- | --- |
| `static FluxIntervalInputFile` | `IntervalVariationManager.getIntervalInputFile(java.lang.String fileName, Fluxome fluxome, MapFormat<LinearComb<java.lang.String>,Interval> mapFormat)`             Returns the flux interval input file with the provided name. |

Uses of Interval in sbrt.shell.text.fba

| Methods in sbrt.shell.text.fba that return Interval | |
| --- | --- |
| `Interval` | `PalssonRxnNodeParser.getFluxBounds(org.w3c.dom.Node reactionNode)`             Returns the flux bounds of the provided reaction node. |
| `Interval` | `BiggRxnNodeParser.getFluxBounds(org.w3c.dom.Node reactionNode)`             Returns the flux bounds of the provided reaction node. |

| Methods in sbrt.shell.text.fba that return types with arguments of type Interval | |
| --- | --- |
| `PipeListFormat<Interval>` | `ConstraintsFileLineFormatV1.getListFormat()`             Returns the format used for lists of values. |
| `PipeListFormat<Interval>` | `ConstraintsFileLineFormatV2.getListFormat()`             Returns the format used for lists of values. |

Uses of Interval in sbrt.shell.text.math

| Methods in sbrt.shell.text.math that return Interval | |
| --- | --- |
| `Interval` | `IntervalFormatV1.parse(java.lang.String interval)`             Parses the provided string and returns its corresponding interval. |
| `Interval` | `BoundedIntervalFormatV1.parse(java.lang.String interval)`             Parses the provided string and returns its corresponding interval. |

| Methods in sbrt.shell.text.math with parameters of type Interval | |
| --- | --- |
| `java.lang.String` | `IntervalFormatV1.format(Interval interval)`             Returns a formatted string representation of the provided interval. |
| `java.lang.String` | `BoundedIntervalFormatV1.format(Interval interval)`             Returns a formatted string representation of the provided interval. |

---


|  |  |  |
| --- | --- | --- |
| **Overview** \| **Package** \| **Class** \| **Use** \| **Tree** \| **Deprecated** \| **Index** \| **Help** | |  |
| PREV   NEXT | **FRAMES**    **NO FRAMES**     **All Classes** |  |


---
